# AI Health Services and Health Satisfaction Across Socioeconomic Groups in South Korea: National Cross-Sectional Study Using an Instrumental Variable Approach

**DOI:** 10.2196/95592

**Published:** 2026-09-15

**Authors:** Hui Zeng, Chuanyang Yu, Yingying Ouyang, Mingzheng Hu

**Affiliations:** 1 School of Social Welfare, Yonsei University, Seoul, Republic of Korea; 2 Party School of Ninghai County Committee of the Communist Party of China, Ningbo, Zhejiang, China; 3 School of Business, Renmin University of China, Beijing, China; 4 Nuffield Department of Primary Care Health Sciences, University of Oxford, Radcliffe Observatory Quarter, Woodstock Road, Oxford, England, OX2 6GG, United Kingdom, 44 7962 630607

**Keywords:** artificial intelligence, AI health, health satisfaction, socioeconomic heterogeneity, South Korea

## Abstract

**Background:**

AI-enabled digital health services are rapidly expanding within health care systems and are expected to improve health management and access to health information. However, rigorous empirical evidence on whether AI health service use is associated with individual health satisfaction remains limited, particularly regarding whether these potential benefits differ across socioeconomic groups.

**Objective:**

This study aims to examine the relationship between AI health service use and health satisfaction and to assess whether this association varies across socioeconomic groups.

**Methods:**

Nationally representative data from the 2024 Digital Divide Survey in South Korea (N=15,000) were analyzed. To address potential endogeneity arising from self-selection and reverse causality, a 2-stage least squares instrumental variable approach was used. Robustness analyses using an alternative sample restriction, an alternative estimation method, and alternative instrumental variable specifications were conducted to assess the robustness of the findings. Subgroup analyses and interaction tests were conducted to assess socioeconomic heterogeneity.

**Results:**

AI health service use was positively and significantly associated with health satisfaction (β=0.739, 95% CI 0.391‐1.088; *P*<.001). The positive association was stronger among men, individuals living outside the capital area, those with lower income, people living alone, and individuals with disabilities.

**Conclusions:**

AI health service use was associated with higher levels of health satisfaction, and this association appeared stronger among several socioeconomically disadvantaged groups. These findings suggest that AI health services may function as complementary health resources and highlight the importance of considering socioeconomic heterogeneity when developing and evaluating AI-enabled health policies.

## Introduction

### Background

Globally, health care systems face persistent challenges, including limited access to care, rising medical costs, and inefficiencies in service delivery [1]. These challenges are particularly pronounced in underresourced settings, where shortages of specialists and advanced medical equipment can lead to delayed diagnosis and adverse health outcomes. Against this backdrop, AI has emerged as a promising technological innovation with the potential to enhance health care accessibility [2,3], efficiency [4,5], and quality [6,7]. By reducing reliance on specialists and improving service delivery, AI health services may expand access to health information and support health management [2,8,9], particularly for populations facing barriers to health care [3]. Accordingly, AI health services are increasingly viewed not only as tools for improving health care delivery but also as technologies with the potential to support population health.

In recent years, health satisfaction has received increasing attention in public health research [10-13] because it captures individuals’ subjective evaluation of their own health and physical condition [14]. Unlike objective health status, which primarily reflects disease or functional limitations, subjective health evaluations integrate individuals’ perceptions of their health experiences and their perceived control over health [15]. These evaluations are also informed by bodily sensations that are directly accessible only to the individual and may reflect subtle physiological changes that are not readily captured by objective clinical indicators, thereby providing a unique source of information about health status [15]. Previous studies have shown that health satisfaction is associated with preventive health behaviors [16], health care utilization [17], and health outcomes [18]. Moreover, subjective health evaluations have been shown to be associated with mortality [19-21]. These findings indicate that health satisfaction captures meaningful dimensions of health that extend beyond objective clinical measures. It therefore offers a useful lens for examining whether AI health service use is related to how individuals perceive their own health.

South Korea provides a particularly informative setting for examining this question. As one of the world’s most digitally advanced societies, South Korea has achieved near-universal internet connectivity and widespread smartphone adoption, creating favorable conditions for the diffusion of AI health services. The Korean government has further promoted AI-Internet of Things–based health care services under its Fourth Industrial Revolution strategy [22], while the Fifth National Health Plan (HP2030) emphasizes reducing health disparities and strengthening health promotion [23]. These technological and policy developments make South Korea an informative setting for examining whether AI health services are associated with improved subjective health-related evaluation. Findings from this context may provide broader insights into the role of AI health services in improving population health in increasingly digitalized societies.

Despite these favorable conditions, empirical evidence examining whether AI health service use is associated with improvements in individual subjective health satisfaction remains limited, particularly in South Korea. Existing studies have primarily focused on efficiency gains [6,24], improvements in health care service delivery [25,26], users’ satisfaction [27], or have provided largely descriptive insights [1,8,28] rather than direct evidence on whether these services translate into meaningful improvements in individual subjective health satisfaction. Furthermore, little is known about whether the health benefits of AI health services are distributed evenly across socioeconomic groups. Addressing these gaps is important for understanding not only the effectiveness of AI health services but also whether their potential benefits are distributed similarly across different population groups.

Against this background, this study had 2 objectives: (1) to examine the association between AI health service use and health satisfaction, and (2) to investigate whether this association differs across socioeconomic groups. To achieve these objectives, nationally representative data from the 2024 Digital Divide Survey in South Korea were analyzed using a 2-stage least squares (2SLS) instrumental variable (IV) approach to address potential endogeneity arising from self-selection and reverse causality.

### Theoretical Framework and Hypotheses

#### AI Health Services and Health Satisfaction

Social Cognitive Theory (SCT) posits that behaviors are shaped through the reciprocal interaction between individuals, their behaviors, and the environment [29]. A central concept of SCT is self-efficacy, which refers to individuals’ beliefs in their ability to perform behaviors necessary to achieve desired outcomes [30]. AI health service use may strengthen self-efficacy for health management by providing personalized health guidance, continuous monitoring, timely feedback, and behavioral reinforcement. As users repeatedly experience successful health management, they may become more confident in their ability to maintain healthy behaviors and cope with health-related challenges. Higher self-efficacy has consistently been associated with health management behaviors [31]. Accordingly, AI health service use may be associated with higher health satisfaction by supporting health-related behaviors and self-management.

H1: AI health service use is positively associated with health satisfaction.

#### Socioeconomic Differences in the Benefits of AI Health Services

The Resource Substitution perspective proposes that the benefits of newly available resources are greater for individuals who possess fewer existing resources because the new resources compensate for preexisting disadvantages [32,33]. Applied to the context of health, AI health services may provide personalized health information, health monitoring, and self-management support that partially substitute for limited health care resources. Individuals with lower socioeconomic resources often face greater barriers to obtaining timely health information and continuous health management. AI health services may therefore provide greater marginal benefits for these populations by expanding access to health support that would otherwise be less available. In contrast, individuals with greater socioeconomic resources often have access to alternative sources of health care support, reducing the incremental benefits of AI health services. Accordingly, the positive association between AI health service use and health satisfaction is expected to be stronger among socioeconomically disadvantaged populations.

H2: The positive association between AI health service use and health satisfaction is stronger among socioeconomically disadvantaged populations.

## Methods

### Data and Study Population

This study used data from the 2024 Digital Divide Survey commissioned by the Ministry of Science and Information and Communications Technology of South Korea and conducted by the National Information Society Agency (NIA). The survey covers both the general population and digitally vulnerable groups, including persons with disabilities, North Korean defectors, low-income individuals, marriage immigrants, and farmers and fishermen. The survey employed a stratified probability proportional sampling method organized by metropolitan local governments. Data were collected through face-to-face interviews between October and December 2024, yielding an initial sample of 15,000 respondents. This cross-sectional study was reported in accordance with the STROBE (Strengthening the Reporting of Observational Studies in Epidemiology) guidelines (Checklist 1).

### Ethical Considerations

The data were obtained from the 2024 Digital Divide Survey (National Approved Statistics number 120017). The survey is an official government-administered statistical program conducted in accordance with national data protection regulations. All data were fully anonymized prior to public release. The dataset was obtained through the NIA, and the applicable terms of data use were followed. The present study involved a secondary analysis of pre-existing data; the authors had no direct contact with respondents and did not collect or record personally identifiable information. Under the relevant legislation of the Republic of Korea, Article 15(2) of the Bioethics and Safety Act and Article 13(1)(3) of its Enforcement Rule provide that research using publicly available information or preexisting data or documents without collecting or recording personally identifiable information may be exempt from institutional review [34]. On this basis, ethics review was not sought for the present analysis.

### Measures

#### Outcome: Health Satisfaction

Health satisfaction was measured using the survey question: “How satisfied are you with your physical and mental health?” Responses were recorded on a 4-point Likert scale: 1=“very dissatisfied,” 2=“somewhat dissatisfied,” 3=“somewhat satisfied,” and 4=“very satisfied.”

#### Exposure: AI Health Services

AI health service use was measured using the survey question: “Have you used health services implemented with AI, such as AI exercise coaches, AI diet management solutions, nutritional information analysis (food image recognition), medical record management (prescription analysis), and insurance company care apps (HELLO, KARE, S-Walking)?” Responses were dichotomized into a binary variable, with AI health service use coded as 1 and no use coded as 0.

#### IV Approach

A key methodological challenge in estimating the association between AI health service use and health satisfaction is potential endogeneity. Individuals who choose to use AI health services may differ systematically from nonusers in unobserved characteristics. In addition, reverse causality may arise if individuals with higher or lower health satisfaction are more likely to adopt AI health services. As a result, conventional regression estimates may be biased.

To address the potential endogeneity, an IV strategy was employed. Similar to previous studies [35,36], respondents were classified into peer groups according to age (<45, 45‐64, ≥65), educational level (primary school, middle school, high school, university or above), household income (high vs low), region of residence (capital vs noncapital area), and disability status (disabled vs nondisabled). For each respondent, the instrument was constructed as the leave-one-out average rate of AI health service use among other respondents within the same socioeconomic-demographic group.

This instrument reflects the degree of exposure to AI health service adoption among socioeconomically and demographically similar peers. According to SCT [29], individuals may acquire new behaviors through observing others in their social environment, particularly those who are perceived as socially similar. Therefore, higher peer adoption may increase individuals’ awareness, familiarity, and perceived acceptability of AI health services, supporting the relevance condition. For the exclusion restriction, the proposed identifying pathway is that peer adoption influences health satisfaction through its effect on the respondent’s own use of AI health services. The instrument was constructed exclusively from AI health service use reported by other respondents within the same peer group, excluding each respondent’s own use, and did not incorporate the respondent’s health satisfaction. Based on this construction and the proposed behavioral pathway, it was assumed that, conditional on the included covariates, peer adoption affects health satisfaction only through the respondent’s own use of AI health services. The IV analysis was therefore conducted under this conditional exclusion restriction.

Finally, to support the exchangeability assumption, all models adjusted for available observable characteristics, including age, gender, educational attainment, household income, residential location, disability status, and living arrangement. These covariates help reduce the potential correlation between the instrument and the error term in the outcome equation. Conditional on these covariates, the remaining variation in peer adoption was assumed to be independent of unobserved determinants of health satisfaction. On this basis, the leave-one-out peer adoption rate was used as the instrument in the IV analysis, conditional on the stated assumptions.

#### Covariates

Several variables were included as covariates to account for potential confounding in the relationship between AI health service use and health satisfaction. Age was treated as a continuous variable. Gender was coded as a binary variable (1=man, 0=woman). Disability status was coded as 1 if the respondent reported having a disability and 0 otherwise. Living arrangement was defined as living alone (1) versus not living alone (0). Residential location was classified as capital area (1) and noncapital area (0). Educational level was categorized into 2 groups: below high school (0) and high school or above (1). Household economic status was measured using monthly household income. According to national statistics, the average monthly household income in South Korea in the fourth quarter of 2024 was approximately 5.2 million KRW (approximately US $3519). Based on this benchmark, households with a monthly income of 6 million KRW (approximately US $4060) or higher were classified as high income (1), and those with income below 6 million KRW (approximately US $4060) were classified as low income (0).

### Statistical Analysis

All analyses were conducted using R software (version 4.4.3; R Foundation for Statistical Computing). First, descriptive analyses were conducted to summarize the demographic and socioeconomic characteristics of the study sample. Categorical variables were reported as frequencies and percentages, while continuous variables were summarized using means and SDs.

Second, the primary association between AI health service use and health satisfaction was estimated using a 2SLS IV approach. In the first stage, individual AI health service use was regressed on the IV. In the second stage, the predicted value of AI health service use from the first stage was included in a linear regression model of health satisfaction. All models adjusted for age, gender, educational attainment, household income, residential location, disability status, and living arrangement. Robustness analyses were conducted using an alternative sample restriction, an alternative estimation method, and alternative IV specifications. In both the primary and robustness analyses, survey sampling weights provided by NIA were incorporated to account for the complex survey design, including the oversampling of digitally vulnerable groups, and to improve the national representativeness of the estimates. In both sets of analyses, cluster-robust standard errors were also estimated based on the peer groups used for IV construction.

Finally, subgroup analyses were conducted to examine potential socioeconomic heterogeneity in the association between AI health service use and health satisfaction. Analyses were stratified by gender, age group (<19, 19‐45, 46‐64, and ≥65 y), residential location (capital area vs noncapital area), living arrangement (living alone vs not living alone), education level (below high school vs high school or above), disability status (with disability vs without disability), and household economic level (low income vs high income). Within each subgroup, the 2SLS model was re-estimated using the same covariate specification as the primary model, with survey sampling weights applied. To account for multiple subgroup comparisons, Benjamini-Hochberg adjusted *P* values were calculated. Interaction terms between AI health service use and each subgroup variable were additionally tested in the full sample to evaluate effect modification. In the IV interaction models, the interaction between AI health service use and each subgroup variable was also instrumented. Specifically, AI health service use was instrumented using the peer adoption instrument, and the interaction term was instrumented using the interaction between the peer adoption instrument and the corresponding subgroup variable.

All reported regression coefficients are unstandardized coefficients with corresponding 95% CIs. All statistical tests were 2-sided, and statistical significance was defined as *P*<.05.

## Results

### Descriptive Characteristics of the Study Population

Table 1 presents the descriptive characteristics of the study population (N=15,000). The mean age of respondents was 49 (SD 18.19) years. Slightly more than half of the participants were men (n=7583, 50.55%), while 7417 (49.45%) were women. Regarding socioeconomic characteristics, 10,676 (71.17%) respondents had completed high school education or above, and 2364 (15.76%) were classified as high-income households. Approximately 2481 (16.54%) participants were living alone. In terms of residential location, 4568 (30.45%) resided in the capital area, whereas 10,432 (69.55%) lived in noncapital regions. Additionally, 2367 (15.78%) respondents reported having a disability. With respect to AI health services, 2541 (16.94%) participants reported use, while 12,459 (83.06%) reported no use. The mean level of health satisfaction was 2.73 (SD 0.74) on a 4-point scale.

**Table 1.** Respondents’ characteristics.

Variables	Participants (N=15,000)
Age, mean (SD)	49 (18.19)
Gender, n (%)	
Man	7583 (50.55)
Woman	7417 (49.45)
Education level, n (%)	
Below high school	4324 (28.83)
High school or above	10,676 (71.17)
Living alone, n (%)	
Yes	2481 (16.54)
No	12,519 (83.46)
Economic level, n (%)	
High income	2364 (15.76)
Low income	12,636 (84.24)
Residence in capital area, n (%)	
Yes	4568 (30.45)
No	10,432 (69.55)
Disability, n (%)	
With disability	2367 (15.78)
Without disability	12,633 (84.22)
AI health services, n (%)	
Used	2541 (16.94)
Not used	12,459 (83.06)
Health satisfaction, mean (SD)	2.73 (0.74)

### Association Between AI Health Services and Health Satisfaction

Table 2 presents the 2SLS regression results with cluster-robust SEs. Because the leave-one-out peer adoption rate could not be calculated for peer groups containing only a single individual, 15 respondents were excluded, resulting in an analytical sample of 14,985 individuals. In the first-stage regression, the IV was strongly associated with AI health service use (β=0.625, SE 0.043, 95% CI 0.541‐0.710; *P*<.001), supporting the relevance of the instrument. The first-stage weak instrument *F* statistic was 340.10, substantially exceeding the critical value of 104.7 [37], indicating that the instrument was sufficiently strong. In the second-stage regression, AI health service use was positively associated with health satisfaction (β=0.739, SE 0.178, 95% CI 0.391‐1.088; *P*<.001). Overall, these findings support Hypothesis 1.

**Table 2.** Two-stage least squares regression estimates of the association between AI health service use and health satisfaction^a^.

Variables	First stage, β^b^ (SE, 95% CI)	*P* value	Second stage, β (SE, 95% CI)	*P* value
AI health service use	—^c^	—	0.739 (0.178, 0.391 to 1.088)	<.001
Instrumental variable	0.625 (0.043, 0.541 to 0.710)	<.001	—	—
Age	−0.001 (0.0002, −0.0014 to −0.0006)	<.001	−0.008 (0.001, −0.010 to −0.007)	<.001
Gender	0.009 (0.009, −0.008 to 0.026)	.31	0.032 (0.012, 0.008 to 0.056)	.009
Educational level	0.037 (0.007, 0.023 to 0.050)	<.001	0.093 (0.033, 0.028 to 0.158)	.005
Economic level	0.037 (0.010, 0.016 to 0.057)	<.001	0.045 (0.029, −0.012 to 0.102)	.13
Living alone	0.019 (0.008, 0.003 to 0.036)	.02	−0.142 (0.024, −0.189 to −0.096)	<.001
Residence in capital area	0.027 (0.006, 0.014 to 0.039)	<.001	−0.014 (0.026, −0.066 to 0.038)	.60
Disability	0.002 (0.008, −0.013 to 0.017)	.82	−0.522 (0.033, −0.586 to −0.458)	<.001

a AI health service use was coded as 1=use and 0=no use. Binary covariates were coded as follows: gender, 1=man and 0=woman; educational level, 1=high school or above and 0=below high school; economic level, 1=high income and 0=low income; living alone, 1=yes and 0=no; residence in capital area, 1=yes and 0=no; disability, 1=with disability and 0=without disability. Age was treated as a continuous variable.

b β denotes the unstandardized regression coefficient.

c Not applicable.

### Robustness Checks

#### Sample Restriction: Korean Nationals Only

To examine whether the main findings were sensitive to sample composition, the analysis was repeated after excluding respondents who did not hold Korean nationality. The same 2SLS model, IV, and clustering strategy as in the primary analysis were retained.

As shown in Table 3, the results for the Korean nationals sample were closely aligned with the primary findings. The IV remained strongly associated with AI health service use in the first stage (β=0.625, 95% CI 0.542‐0.709; *P*<.001), with a weak-instrument *F* statistic of 342.20, substantially exceeding the conventional threshold of 104.7. In the second stage, AI health service use continued to be positively associated with health satisfaction (β=0.732, 95% CI 0.381‐1.083; *P*<.001). After excluding 320 non-Korean respondents, an additional 15 respondents belonging to peer groups containing only a single individual were removed because the leave-one-out peer adoption rate could not be calculated, resulting in an analytical sample of 14,665 respondents. These findings suggest that the main results are robust to restricting the analysis to Korean nationals.

**Table 3.** Results of various robustness tests^a^.

Variables	Sample restriction		Alternative estimation method		Alternative instrumental variable specifications			
	Korean nationals only (2SLS^b^)		Ordered-outcome specification (2SRI^c^)		Leave-one-region-out IV^d^ (2SLS)		Split-sample cross-fitted IV (2SLS)	
	First stage	Second stage	First stage	Second stage	First stage	Second stage	First stage	Second stage
AI health service use	—^e^	0.732^f^ (0.381-1.083)	—	1.295^f^ (0.716-1.875)	—	1.203^f^ (0.638-1.768)	—	0.728^f^ (0.366-1.089)
Instrumental variable	0.625^f^ (0.542-0.709)	—	0.625^f^ (0.541-0.710)	—	0.450^f^ (0.267-0.634)	—	0.533^f^ (0.444-0.622)	—
First-stage *F* statistic	342.20	—	340.10	—	119.05	—	137.02	—
Control variables	YES	YES	YES	YES	YES	YES	YES	YES
Observations	14,665	14,665	14,985	14,985	14,994	14,994	14,963	14,963

a Numbers in parentheses are 95% CIs. Control variables included age, gender, educational attainment, household income, residential location, disability status, and living arrangement. “YES” indicates that the corresponding controls were included.

b 2SLS: 2-stage least squares.

c 2SRI: 2-stage residual inclusion.

d IV: instrumental variable.

e Not applicable.

f *P*<.001.

#### Alternative Model Strategy: Ordered-Outcome Specification

Because the dependent variable, health satisfaction, is measured on a 4-point scale, the main analysis based on a linear IV model was re-estimated using a 2-stage residual inclusion IV ordered probit model as a robustness check. The IV was constructed using the same peer-group approach as in the primary analysis.

As shown in Table 3, the IV remained strongly associated with AI health service use in the first stage (β=0.625, 95% CI 0.541‐0.710; *P*<.001), with a weak-instrument *F* statistic of 340.10. In the second-stage IV ordered probit model, AI health service use remained positively associated with health satisfaction (β=1.295, 95% CI 0.716‐1.875; *P*<.001). As in the main analysis, respondents belonging to peer groups containing only a single individual were excluded because the leave-one-out peer adoption rate could not be calculated, resulting in an analytical sample of 14,985 individuals.

Although the estimated coefficient from the IV ordered probit model differed in magnitude from that of the linear 2SLS model, both models indicated a positive and statistically significant association between AI health service use and health satisfaction. The IV ordered probit coefficient is expressed on the latent-response scale underlying the ordered outcome, whereas the linear 2SLS coefficient is expressed in units of the observed 4-point health satisfaction measure; therefore, the 2 coefficients are not directly comparable in magnitude. Thus, the difference in coefficient magnitude should be interpreted in light of differences in model specification and coefficient scale rather than differences in the substantive conclusions, supporting the robustness of the primary findings with respect to the direction and statistical significance of the association.

#### Alternative Instrument Construction: Leave-One-Region-Out Peer Adoption Instrument

To further assess the robustness of the identification strategy, the IV was reconstructed using a leave-one-region-out approach, and the resulting model was estimated using 2SLS. Specifically, peer groups were redefined using age group, education level, income level, and disability status, excluding the capital-area indicator used in the primary instrument. For each respondent, the instrument was calculated as the average AI health service use among individuals belonging to the same demographic-socioeconomic peer group but residing outside the respondent’s own geographic region. Geographic regions were defined according to the 7 regional classifications available in the survey (Seoul, Gyeongin, Chungcheong, Honam, Gyeongbuk, Gyeongnam, and Other). This approach reduces the possibility that local contextual factors simultaneously influence both peer adoption and individual health satisfaction.

The alternative IV significantly predicted AI health service use in the first stage (β=0.450, 95% CI 0.267‐0.634; *P*<.001), and the weak-instrument statistic remained sufficiently large (*F*=119.05), indicating that instrument relevance was preserved. In the second-stage 2SLS model, AI health service use remained positively and statistically significantly associated with health satisfaction (β=1.203, 95% CI 0.638‐1.768; *P*<.001). The leave-one-region-out 2SLS estimate was larger than the primary 2SLS estimate (β=0.739). The larger estimate may reflect differences in peer-group composition and in the source of identifying variation generated by excluding respondents from the same geographic region, while the direction and statistical significance of the association remained consistent across the 2 specifications. Because 6 observations could not be assigned a leave-one-region-out instrument, the analytical sample consisted of 14,994 respondents.

#### Alternative Instrument Construction: Split-Sample Cross-Fitted IV

Finally, a split-sample cross-fitting procedure was implemented to further alleviate concerns that the peer-based IV might mechanically reflect an individual’s own contribution to the peer adoption rate. The association was then reestimated using the resulting cross-fitted instrument within the same 2SLS framework as the primary analysis. The full sample was randomly divided into 2 approximately equal subsamples. Using the same peer-group definition as in the primary analysis, average AI health service use was calculated separately within each subsample. Respondents in one subsample were then assigned the peer-adoption rate estimated from the opposite subsample, ensuring that each individual’s IV was generated entirely from observations not used to estimate their own outcome.

As shown in Table 3, the cross-fitted IV remained a strong predictor of AI health service use in the first stage (β=0.533, 95% CI 0.444‐0.622; *P*<.001). The first-stage *F* statistic was 137.02, well above the conventional threshold for weak instruments. In the second-stage 2SLS model, AI health service use remained positively and statistically significantly associated with health satisfaction (β=0.728, 95% CI 0.366‐1.089; *P*<.001). During construction of the cross-fitted instrument, 37 observations belonged to peer groups represented by only one individual in one of the split samples and therefore could not be assigned a valid instrument. These observations were excluded, leaving a final analytical sample of 14,963 respondents.

### Subgroup and Interaction Analyses

**Figure 1.**
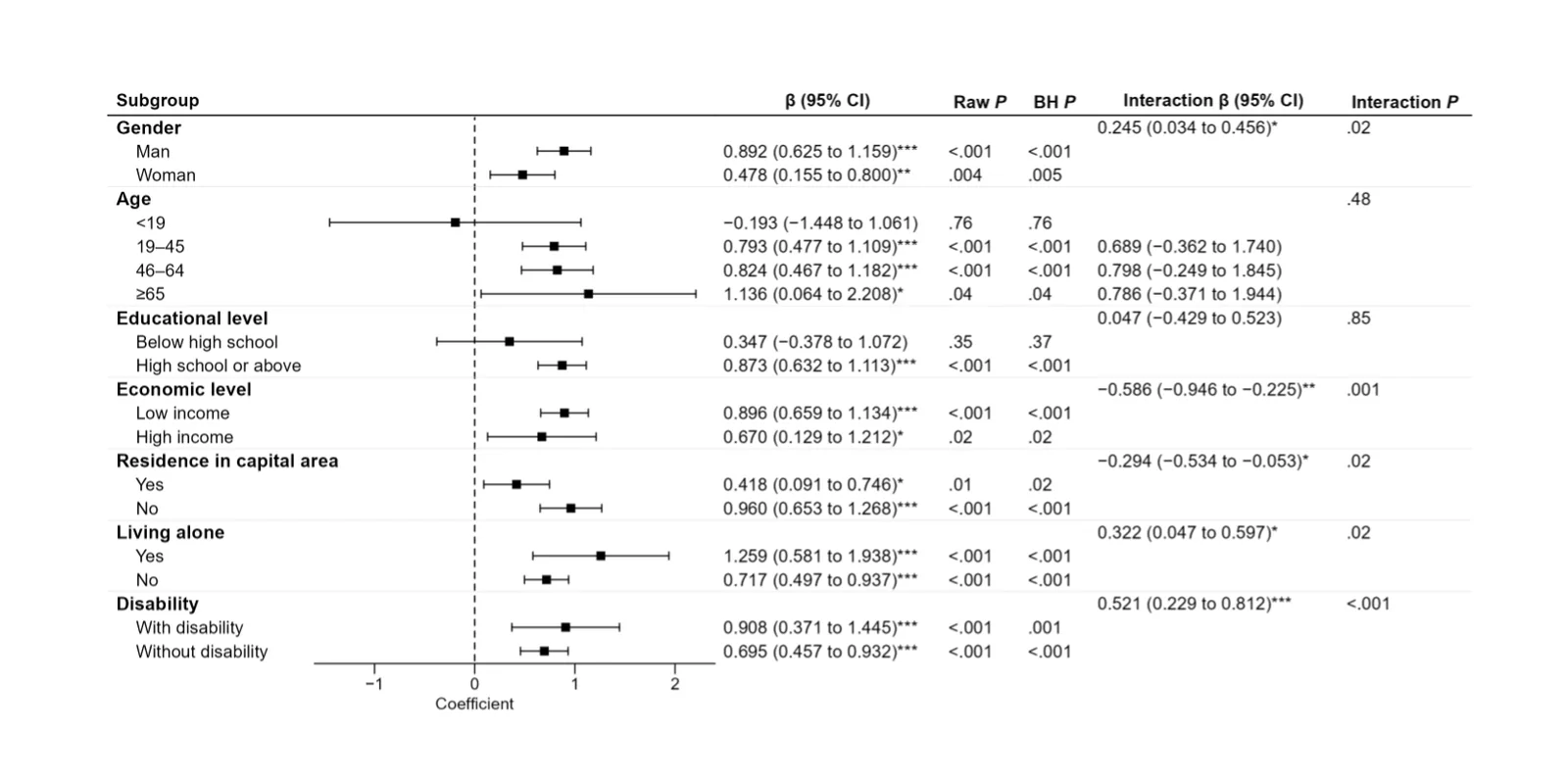
Subgroup-specific estimates and interaction tests. β denotes the unstandardized regression coefficient; BH: Benjamini-Hochberg. Subgroup-specific coefficients were obtained from separate stratified 2-stage least squares models. Raw *P* values refer to subgroup-specific estimates, and BH *P* values refer to Benjamini-Hochberg adjusted *P* values for multiple subgroup comparisons. ^***^P<.001, ^**^P<.01, ^*^P<.05.

Figure 1 summarizes subgroup-specific estimates and formal interaction tests for the association between AI health service use and health satisfaction. Overall, AI health service use was positively associated with health satisfaction across most subgroups, and most subgroup-specific associations remained statistically significant after Benjamini-Hochberg correction. However, the subgroup-specific estimates were not statistically significant among respondents younger than 19 years or among those with below-high-school education.

Formal interaction tests showed significant subgroup differences by gender, household economic status, residential location, living arrangement, and disability status. The positive association was stronger among men than women (β=0.892 vs 0.478; *P* for interaction=.02), low-income than high-income respondents (β=0.896 vs 0.670; *P* for interaction=.001), respondents living outside the capital area than those living in the capital area (β=0.960 vs 0.418; *P* for interaction=.02), individuals living alone than those not living alone (β=1.259 vs 0.717; *P* for interaction=.02), and respondents with disabilities than those without disabilities (β=0.908 vs 0.695; *P* for interaction <.001).

No statistically significant interactions were observed for age (*P* for interaction=.48) or educational level (*P* for interaction=.85), indicating that the association between AI health service use and health satisfaction did not appear to differ significantly across these subgroups. Overall, these findings provided partial support for Hypothesis 2. The interactions for household economic status, residential location, living arrangement, and disability status were consistent with the Resource Substitution perspective. A significant gender interaction was also observed, although the stronger association among men was not directly predicted by Hypothesis 2, while age and educational attainment showed no evidence of effect modification.

## Discussion

### Principal Findings

This study examined whether AI health service use was associated with health satisfaction and whether this association differed across socioeconomic groups using nationally representative data from South Korea and an IV approach to address potential endogeneity. The findings demonstrated that AI health service use was positively associated with health satisfaction. This finding extends previous research that has primarily focused on the technological capabilities of AI health care, including improvements in health care efficiency, diagnostic performance, and service delivery [24], by showing that AI health service use was also positively associated with individuals’ health satisfaction.

Another important finding of this study is that the association between AI health service use and health satisfaction was not uniform across population groups. The positive association was significantly stronger among individuals with lower household income, those living outside the capital area, people living alone, and individuals with disabilities. These findings are broadly consistent with the Resource Substitution perspective, which suggests that newly available resources may generate greater benefits among individuals with fewer existing resources [32]. Although previous discussions of digital health inequalities have emphasized that disadvantaged populations may be less likely to access and adopt emerging technologies [38-40], the present findings suggest a more nuanced picture. While inequalities in technology adoption may persist, those who do adopt these services may derive greater health-related benefits when alternative health care resources are limited. These findings add to existing discussions of digital health equity [2,3].

The interaction tests did not identify significant variation in the association by age or educational attainment. Although men are not conventionally characterized as socioeconomically disadvantaged, the stronger association observed among men may be interpreted through a broader application of the Resource Substitution perspective that considers differences in existing health-information resources. Previous research in South Korea has found that women are more likely than men to use the internet for health information [41] and report higher levels of health literacy in several domains [42]. Women may therefore already draw on a broader range of health-information resources, reducing the additional value provided by AI health services. In contrast, AI health services may offer men an additional, self-directed channel for obtaining health information and managing their health, resulting in a greater marginal association with health satisfaction.

The observed positive association in this study may be explained through several potential pathways. First, AI health services may promote health self-management by providing personalized exercise coaching [43,44], dietary guidance [45,46], personalized health reminders [47], and continuous monitoring [48]. Behavioral improvements facilitated by AI health services may increase health management self-efficacy, ultimately contributing to higher health satisfaction. In addition, AI health services provide continuous access to personalized health information and immediate feedback, which may alleviate health-related anxiety and improve individuals’ health satisfaction. These behavioral and psychological processes may help explain the positive association.

This study makes 3 contributions to the existing literature. First, by conceptualizing health satisfaction as a user-centered, self-reported appraisal of physical and mental health, this study broadens the evaluation of AI health services beyond conventional indicators such as technological performance [7,49], diagnostic accuracy [24,50], and operational efficiency [24,51]. Second, the heterogeneity findings further refine the applicability of the Resource Substitution [33] perspective in the context of digital health. The larger estimated associations observed among lower-income respondents, residents outside the capital area, people living alone, and people with disabilities are consistent with this perspective, suggesting that AI health services may have greater marginal value when conventional health information, social support, or health-management resources are relatively limited. Third, this study provides new empirical evidence from South Korea as a highly digitalized critical case. Even in a setting characterized by advanced digital infrastructure, widespread connectivity, and strong policy support for AI health services, the association between AI health service use and health satisfaction remained socially patterned. This suggests that individuals’ ability to translate technological access into meaningful health-management support continues to be shaped by complementary socioeconomic and social resources.

Several limitations should also be acknowledged. First, although the IV approach and multiple robustness analyses strengthened confidence in the findings, the cross-sectional design prevents definitive conclusions regarding long-term causal effects. Second, health satisfaction was measured using a self-reported indicator and may be influenced by reporting differences across individuals. Future research should incorporate objective health indicators to provide a more comprehensive assessment of health outcomes. Third, due to the limitations of the available data, the survey did not ask respondents to report the specific types of AI health services they used. Therefore, this study could not further examine whether different types, frequencies, or intensities of AI health service use were differently associated with health satisfaction. Finally, although positive associations were observed across the primary and alternative IV specifications, the exclusion restriction cannot be directly tested. Peer adoption may still capture unobserved group-level characteristics, such as health literacy or shared attitudes toward technology, which may also be related to health satisfaction. Therefore, the findings should be interpreted as reflecting associations rather than definitive causal relationships. Future research may consider using more rigorous research designs, such as randomized controlled trials, to validate the causal effects of AI health service use on health satisfaction.

These findings also have important implications for AI health policy. In promoting AI health services, policymakers may consider paying greater attention to socioeconomically disadvantaged groups, including individuals with lower income, residents outside the capital area, people living alone, and individuals with disabilities. Targeted strategies, such as improving affordability, accessibility, and digital support for these groups, may facilitate their effective use of AI health services and help them derive greater health-related value from these technologies.

Nevertheless, these findings should not be interpreted as suggesting that AI health services are universally beneficial. Previous studies have shown that large language models and AI health applications may generate incomplete or misleading health information, particularly when responding to complex clinical questions [52,53]. Therefore, AI health services should be viewed as complementary tools that support, rather than replace, professional health care.

### Conclusions

AI health service use was positively associated with health satisfaction, with stronger associations observed among men, individuals with lower income, residents outside the capital area, people living alone, and individuals with disabilities. These findings suggest that AI health services may serve as complementary health resources for populations with relatively limited health and social resources. More broadly, these findings highlight the importance of evaluating AI health services not only in terms of technological performance but also in terms of their potential contributions to subjective health outcomes and the distribution of these benefits across socioeconomic groups. These insights may help inform the design and implementation of AI-enabled health policies that better reflect the diverse needs of different population groups.

## Supplementary material

10.2196/95592 Checklist 1 STROBE checklist for cross-sectional studies.

## Abbreviations

2SLS: 2-stage least squares
IV: instrumental variable
NIA: National Information Society Agency
SCT: Social Cognitive Theory
STROBE: Strengthening the Reporting of Observational Studies in Epidemiology
